# Multimodal Analysis of *SCN1A* Missense Variants Improves Interpretation of Clinically Relevant Variants in Dravet Syndrome

**DOI:** 10.3389/fneur.2019.00289

**Published:** 2019-03-28

**Authors:** Marina C. Gonsales, Maria Augusta Montenegro, Paula Preto, Marilisa M. Guerreiro, Ana Carolina Coan, Monica Paiva Quast, Benilton S. Carvalho, Iscia Lopes-Cendes

**Affiliations:** ^1^Department of Medical Genetics and Genomic Medicine, School of Medical Sciences, The Brazilian Institute of Neuroscience and Neurotecnology, University of Campinas, Campinas, Brazil; ^2^Department of Neurology, School of Medical Sciences, The Brazilian Institute of Neuroscience and Neurotecnology, University of Campinas, Campinas, Brazil; ^3^Department of Statistics, Institute of Mathematics, Statistics and Scientific Computing, The Brazilian Institute of Neuroscience and Neurotecnology, University of Campinas, Campinas, Brazil

**Keywords:** epileptic encephalopathy, ion channel gene defects, clinical genetic testing, variants of uncertain significance, VUS

## Abstract

**Objective:** We aimed to improve the classification of *SCN1A* missense variants in patients with Dravet syndrome (DS) by combining and modifying the current variants classification criteria to minimize inconclusive test results.

**Methods:** We established a score classification workflow based on evidence of pathogenicity to adapt the classification of DS-related *SCN1A* missense variants. In addition, we compiled the variants reported in the literature and our cohort and assessed the proposed pathogenic classification criteria. We combined information regarding previously established pathogenic amino acid changes, mode of inheritance, population-specific allele frequencies, localization within protein domains, and deleterious effect prediction analysis.

**Results:** Our meta-analysis showed that 46% (506/1,101) of DS-associated *SCN1A* variants are missense. We applied the score classification workflow and 56.5% (286/506) of the variants had their classification changed from VUS: 17.8% (90/506) into “pathogenic” and 38.7% (196/506) as “likely pathogenic.”

**Conclusion:** Our results indicate that using multimodal analysis seems to be the best approach to interpret the pathogenic impact of *SCN1A* missense changes for the molecular diagnosis of patients with DS. By applying the proposed workflow, most DS related *SCN1A* variants had their classification improved.

## Introduction

Recent scientific and technical breakthroughs have allowed for the increasing use of genetic testing in clinical practice, providing more precise diagnoses and having significant prognostic and treatment implications (1, 2). However, interpreting the clinical significance of genetic variants found in molecular tests can still be challenging, particularly for variants of uncertain significance (VUS) (3). A straightforward answer for clinicians regarding these variants is often not possible, thus posing an important difficulty in risk assessment and proper genetic counseling (1).

Currently, *SCN1A*, which encodes the neuronal voltage-gated sodium channel Nav1.1, is considered one of the most relevant epilepsy-related genes in the clinical setting (1, 4). Patients with Dravet syndrome (DS) possess the majority of *SCN1A* variants identified to date, with variants detected in 70–80% of these patients (4, 5). DS is an epileptic encephalopathy characterized by early onset febrile tonic clonic seizures followed by myoclonic jerks, atypical absences, and complex focal seizures and is highly resistant to treatment with antiepileptic drugs (6, 7).

Because determining the pathogenicity of VUS is crucial to interpreting their clinical significance (3), we aimed to improve the classification of *SCN1A* missense variants identified in patients with DS by combining and modifying the criteria for classifying variants proposed by the American College of Medical Genetics and Genomics (ACMG) and the Association for Molecular Pathology (AMP) (3). To do so, we established a score classification workflow based on evidence of pathogenicity to adapt the classification of potentially deleterious DS-related *SCN1A* missense variants. In addition, we compiled the *SCN1A* variants reported in individuals with DS from the literature and our cohort and assessed the proposed pathogenic classification criteria.

## Materials and Methods

### Patients

Our molecular study included 21 Brazilian probands with DS, of whom two were monozygotic twins and the others were unrelated. Patients were regularly followed in an outpatient epilepsy clinic and fulfilled the clinical criteria for DS according to the International League Against Epilepsy (ILAE) guidelines (8, 9). The clinical protocol included a complete neurological examination, analysis of seizure history, EEG recordings, and neuroimaging. All parents signed an informed consent form prior to entering the study, which was approved by the Research Ethics Committee of the University of Campinas (UNICAMP), Brazil, under the number 032/2,006. Detailed clinical features of 12 patients have been previously published (10).

### Mutation Screening

DNA samples for each patient and their parents, when available, were obtained from peripheral blood lymphocytes by standard procedures (11). All 26 coding exons and intron-exon boundaries of *SCN1A* were amplified by PCR (primer sequences available upon request) and Sanger sequenced by capillary electrophoresis in an ABI 3500xL Genetic Analyzer using the BigDye® Terminator Cycle Sequencing Kit (Thermo Fisher Scientific, Waltham, MA, USA). Sequence variants were described according to the conventional nomenclature (12) based on the full-length *SCN1A* isoform (GenBank AB093548) and deposited in a public genomic database of epileptic encephalopathies^1^.

To detect copy number variations within *SCN1A*, we performed multiplex ligation-dependent probe amplification (MLPA) assays using the SALSA MLPA P137-2 kit (MRC-Holland, Amsterdam, Netherlands). MLPA reactions were conducted according to the manufacturer's instructions, and the fragments were separated in an ABI 3500xL Genetic Analyzer. Data analysis was performed using ABI Gene Mapper (Thermo Fisher Scientific) and Coffalyser (MRC-Holland) software.

### Score Classification Workflow for Pathogenic Variants

We revised and modified the ACMG/AMP pathogenic rules to determine which criteria apply to our framework and established a score classification workflow for DS-related *SCN1A* missense variants based on five key aspects, addressed as comprehensive questions. We attributed different points to the answers according to the correspondent evidence of pathogenicity. Based on the revision of rules adaptation made by Kelly et al. (13), we also removed criteria from the current guidelines that are not pertinent to our framework, such as the ones that do not involve missense mutations or that are exclusively applied to a recessive inheritance.

To expand the data on *SCN1A* variants in patients with DS and improve our understanding on the aspects addressed in the classification workflow proposed, we performed an updated meta-analysis of published studies and then investigated the proposed pathogenic classification criteria in the overall sample.

### Meta-Analysis

A literature search was performed using the online search engine PubMed^2^ for the terms “*SCN1A* AND (Dravet OR epilepsy OR encephalopathy)” up to May 2018. We conducted a systematic review of *SCN1A* variants that were publicly available. When necessary, variants were renamed according to the full-length *SCN1A* isoform (GenBank AB093548). We computed the number of individuals that harbor each variant and tried our best to eliminate duplicates and individuals reported more than once in different papers.

### Assessment of the Pathogenic Classification Criteria

Using the *SCN1A* missense variants in DS compiled from the literature and those identified in this study, we assessed the pathogenic classification criteria addressed in the score classification workflow, focusing on five key aspects:

#### Previously Established Pathogenic Amino Acid Changes

For each amino acid change reported, we investigated if it had been previously established as pathogenic in other studies, caused either by the same nucleotide change or by another. Moreover, we compiled information on functional studies demonstrating the deleterious effect of the amino acid changes.

#### Inheritance of Variants

We gathered all the information available on the mode of inheritance of the *SCN1A* variants and assessed whether they were present or absent (*de novo*) in the parents. We also evaluated if the variants co-segregate with the disease in multiple affected family members.

#### Allele Frequency in Population Databases

First, we estimated the maximum expected allele frequency for DS-associated variants in the general population, calculating the threshold with the formula proposed by Whiffin et al. (14), available as an “Allele Frequency App”^3^. Because causal variants for most Mendelian disorders are expected to be rare, we established a stringent allele frequency threshold accounting for disease prevalence, genetic and allelic heterogeneity, inheritance mode and penetrance (14). We used as disease prevalence 1/20,000 (15), genetic heterogeneity of 1% since the most common variant accounts for almost 1% of DS cases, and overall penetrance of 77% for missense variants (16). The threshold for the maximum credible population allele frequency was 3.25e-07.

Then, we assessed the presence of *SCN1A* missense mutations in databases of individuals from different populations: BipMed^4^, NHLBI Exome Sequencing Project (ESP)^5^, gnomAD^6^, and 1,000 Genomes^7^.

#### The Predominance of Amino Acid Changes in Specific Protein Regions

To test whether amino acid changes associated with DS were predominant in specific segments of the Nav1.1 protein, we performed a permutation test. We defined 14 different protein regions: the segments of each domain (S1 to S6), the regions between segments (S1–S2, S2–S3, S3–S4, S4–S5, S5–S6), the regions between domains (S), and the two terminal regions (N- and C- terminal). The total number of alterations per amino acid position was exchanged 5,000 times and, for each permutation, the sum of alterations per region was calculated. The *P*-value for each protein region was calculated as the proportion of permutations in which the number of alterations was equal to or higher than the one observed for that region. We used Fisher's test to verify the joint significance of *P*-values at the 5% level. Once a significant signal was detected, the *P*-values per region were adjusted using the false discovery rate (FDR).

#### Deleterious Prediction Analysis

To predict the impact on protein function of the *SCN1A* missense mutations previously described and the ones found in our cohort, we employed 13 of the 16 computer algorithms recommended by the ACMG/AMP guidelines: FATHMM (17)^8^, Condel (18)^9^, MutationTaster (19)^10^, PANTHER (20)^11^, SNPs&GO (21)^12^, MutPred2 (22)^13^, PROVEAN (23)^14^, CADD (24)^15^, PolyPhen2 (25)^16^, MutationAssessor (26)^17^, SIFT (27)^18^, Align GVGD (28)^19^, PhD-SNP (29)^20^. We choose the algorithms that have an online interface, that analyze specific variants instead of giving scores for each amino acid in the protein, and that do not require 3D structure, as it is not available for Nav1.1. The prediction analyses are based on conservation of orthologous sequences and the physiochemical differences between the wild-type amino acid and the one resulting from the missense substitution.

Finally, we applied the score classification workflow and calculated the scores for all DS-related missense *SCN1A* variants.

## Results

### Mutation Screening

*SCN1A* mutation screening revealed potentially pathogenic variants in 17 of the 21 patients with DS from our cohort (81%), two of whom were monozygotic twins and presented the same alteration. All 16 variants were present in the heterozygous form and are shown in Table 1, along with their predicted protein repercussions. More detailed clinical data from patients 1, 2, 5–9, 11–13 have been previously published (10). All variants have been deposited and are publicly available at http://bipmed.iqm.unicamp.br/epileptic-encephalopathy.

**Table 1:** Sixteen predicted deleterious *SCN1A* variants found in our cohort of 21 patients with Dravet syndrome.

**Mutation type**	**DNA change**	**Amino acid change**	**Seizure onset (months)**	**Febrile seizure (temperature, ^**°**^C)**	***Status epilepticus***	**Family history of seizures**
Missense	c.829T>C	p.C277R	4	Yes (37.5–38)	Yes	No
	c.971A>C	p.H324P	3	Yes (37.3)	No	No
	c.2360T>G	p.M787R	5	Yes (37.2)	NI	NI
	c.4093G>T	p.G1365C	2	Yes (NI)	NI	No
	c.5179G>T	p.D1727Y	3	Yes (38)	Yes	No
	c.5434T>C	p.W1812R	5	Yes (37–37.5)	Yes	No
Splice site	IVS2+1A>G	–	3	Yes (NI)	Yes	Yes
	IVS4+1G>A	–	4	Yes (37.5)	No	No
	IVS8+3G>T	–	7	Yes (37–37.3)	No	Yes
	IVS21+1G>A	–	4	Yes (NI)	NI	NI
Frameshift	c.1242delA	p.I415X	6	Yes (38)	Yes	Yes
	c.3719_3720insGATA	p.I1240fsX1244	4	Yes (37.8)	Yes	No
	c.5329delG	p.V1777fsX1778	3	Yes (38)	Yes	Yes
In-frame	c.296_313delTCTTCCGGTTCAGTGCCA	p.I99_A104del	6	Yes (37)	NI	Yes
	c.296_313delTCTTCCGGTTCAGTGCCA	p.I99_A104del	8	Yes (37)	NI	Yes
	c.5489_5491delAGT	p.Q1830_F1831delinsL	NI	NI	NI	NI
Nonsense	c.5177G>A	p.W1726X	5	Yes (NI)	NI	NI

*All of these variants have been deposited and are publicly available at http://bipmed.iqm.unicamp.br/epileptic-encephalopathy. NI, not informed*.

MLPA analysis did not reveal any copy number variants in *SCN1A* in the other patients with DS apart from the 18-base-pair deletion found in the monozygotic twins. None of the potentially deleterious mutations identified in our cohort of patients with DS were observed in their parents.

### Score Classification Workflow

**Figure 1:**
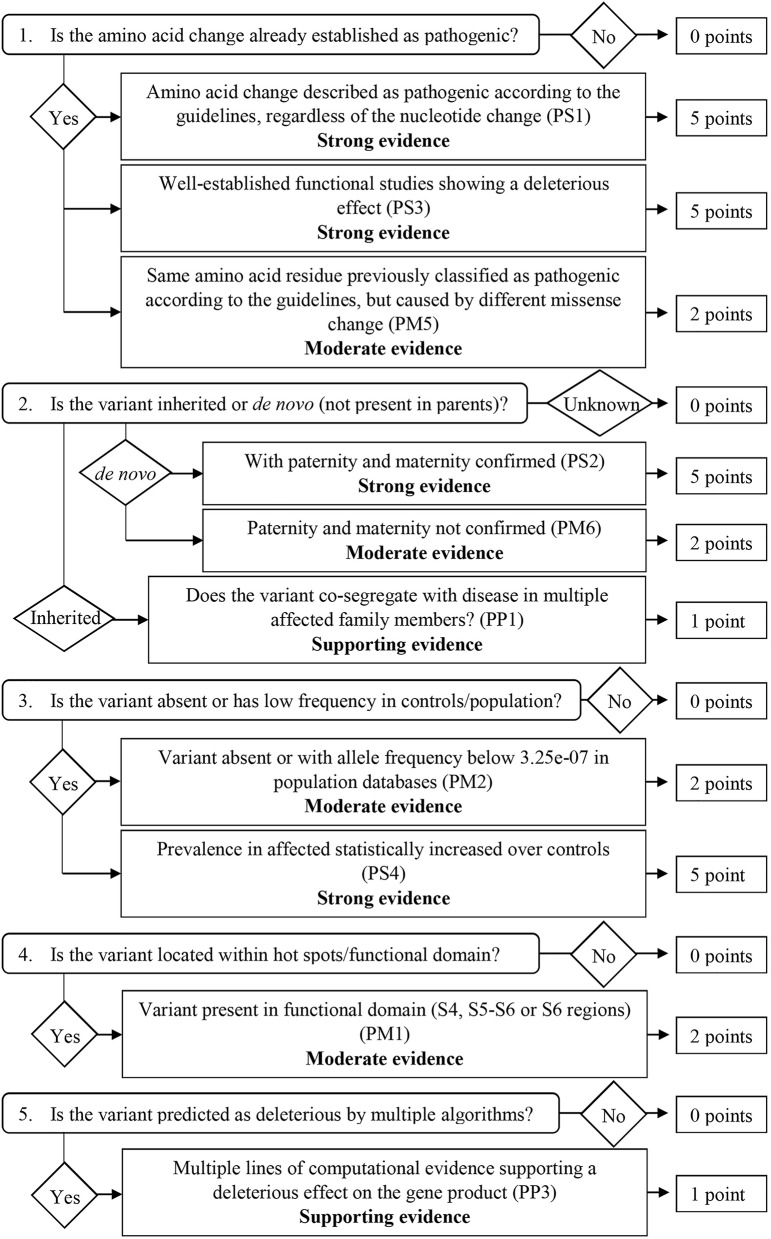
Classification workflow used in the present study.

From the 16 ACMG/AMP pathogenic criteria, we considered six not applicable to our framework (PVS1, PM3, PM4, PP2, PP4, and PP5). We used the remaining ten criteria into a classification workflow structured in five questions, leading to different scores (Figure 1). We attributed five points for strong evidence of pathogenicity, two points for moderate evidence, and one point for supporting evidence. These scores meet the ACMG/AMP guidelines for combining criteria to classify sequence variants adapted to our framework, as shown in Table 2. Thus, a missense variant is considered “pathogenic” when the score is equal or higher than 10 points, and “likely pathogenic” when the score ranges from six to nine points.

**Table 2:** Modified proposed guidelines for combining different criteria to classify *SCN1A* missense variants in the context of the molecular diagnosis of patients with Dravet syndrome variants into “pathogenic” or “likely pathogenic” and correspondent scores.

**Classification**	**Evidences of pathogenicity**	**Score**
Pathogenic	≥2 Strong	≥10
	1 Strong + 3 or 4 Moderate	11 or 13
	1 Strong + 2 Moderate + 2 Supporting	11
Likely pathogenic	1 Strong + 1 or 2 Moderate	7 or 9
	1 Strong + 2 Supporting	7
	3 or 4 Moderate	6 or 8
	2 Moderate + 2 Supporting	6

### *SCN1A* Meta-Analysis

The compilation of *SCN1A* variants reported in the literature, in addition to those identified in the present work, revealed a total of 1,101 potentially deleterious nucleotide variants in patients with DS (Table S1). Of these, 46.0% (506/1,101) are missense mutations; 27.0% (297/1,101), frameshift mutations; 13.0% (144/1,101), nonsense mutations; 11.0% (121/1,101), splice site mutations; and 3.0% (33/1,101) are in-frame insertions or deletions.

When considering the total number of variants, including those found in multiple individuals with Dravet syndrome, the proportion remain similar: 45.0% (700/1,555) missense mutations; 22.0% (342/1,555) frameshift mutations; 19.6% (305/1,555) nonsense mutations; 11.1% (172/1,555) splice site mutations; 2.3% (36/1,555) in-frame insertions or deletions.

### Assessment of the Pathogenic Classification Criteria

#### Previously Established Pathogenic Amino Acid Changes

We evaluated which *SCN1A* variants had been previously reported as associated with DS and observed that 18.2% (92/506) of the missense variants were identified in more than one individual with DS. Furthermore, 10.3% (52/506) were also found in individuals with other phenotypes: mostly in borderline DS/severe myoclonic epilepsy of infancy borderline (SMEB), comprising 5.1% (26/506), and genetic epilepsy with febrile seizures plus (GEFS+) or febrile seizures (FS), with 2.8% (14/506). Four variants encompass the same amino acid change but are caused by a different nucleotide substitution. Moreover, 42.7% (216/506) of the variants result in the change of an amino acid residue where a different change has been reported.

Regarding functional data, we found few studies evaluating the deleterious effect of the amino acid changes reported in DS patients. In fact, only 4.8% (24/502) of the amino acid changes described in DS have been functionally tested, and they all showed an impact on the Nav1.1 function.

#### Inheritance of Variants

From the data available on the mode of inheritance of the *SCN1A* missense variants associated with DS, we observed that 65.0% (329/506) are reported as *de novo*, 51.4% (260/506) of them found exclusively *de novo*, without being present in the parents of any individuals described with that variant. In addition, of the 260 *de novo SCN1A* missense variants reported in the literature, 253 (97.3%) were found exclusively in patients with DS. Moreover, the missense variants that were also found in GEFS+ and other phenotypes are more frequently inherited/familial (22/52, 42.3%) rather than exclusively *de novo* (7/52, 13.5%).

As for variants reported as familial or inherited (maternal, paternal, biparental or not specified), they comprise 13.0% (66/506) of the missense variants. However, 14 of them are also reported as *de novo* in other individuals (2.8% of all missense). Furthermore, we found that a high number of missense variants do not have their mode of inheritance determined, with 24.9% (126/506) of all missense variants exclusively reported as of unknown inheritance.

#### Allele Frequency in Population Databases

We identified 29 of the 506 variants (5.7%) in four different databases, all of them with allele frequency higher than 3.25e-07 in the populations represented (Table S2).

Two variants (c.1811G>A and c.5782C>G) were present in all four databases, and they were the only DS-related variants found in the Brazilian population database (www.bipmed.org), both with allele frequencies of approximately 0.5%. In addition, the variant c.1811G>A was the only one with an allele frequency higher than 0.05% in the Ashkenazi Jewish and South Asian populations. In fact, the frequency of this variant reaches 0.98 and 2.08% in two different South Asian populations.

#### The Predominance of Amino Acid Changes in Specific Protein Regions

**Figure 2:**
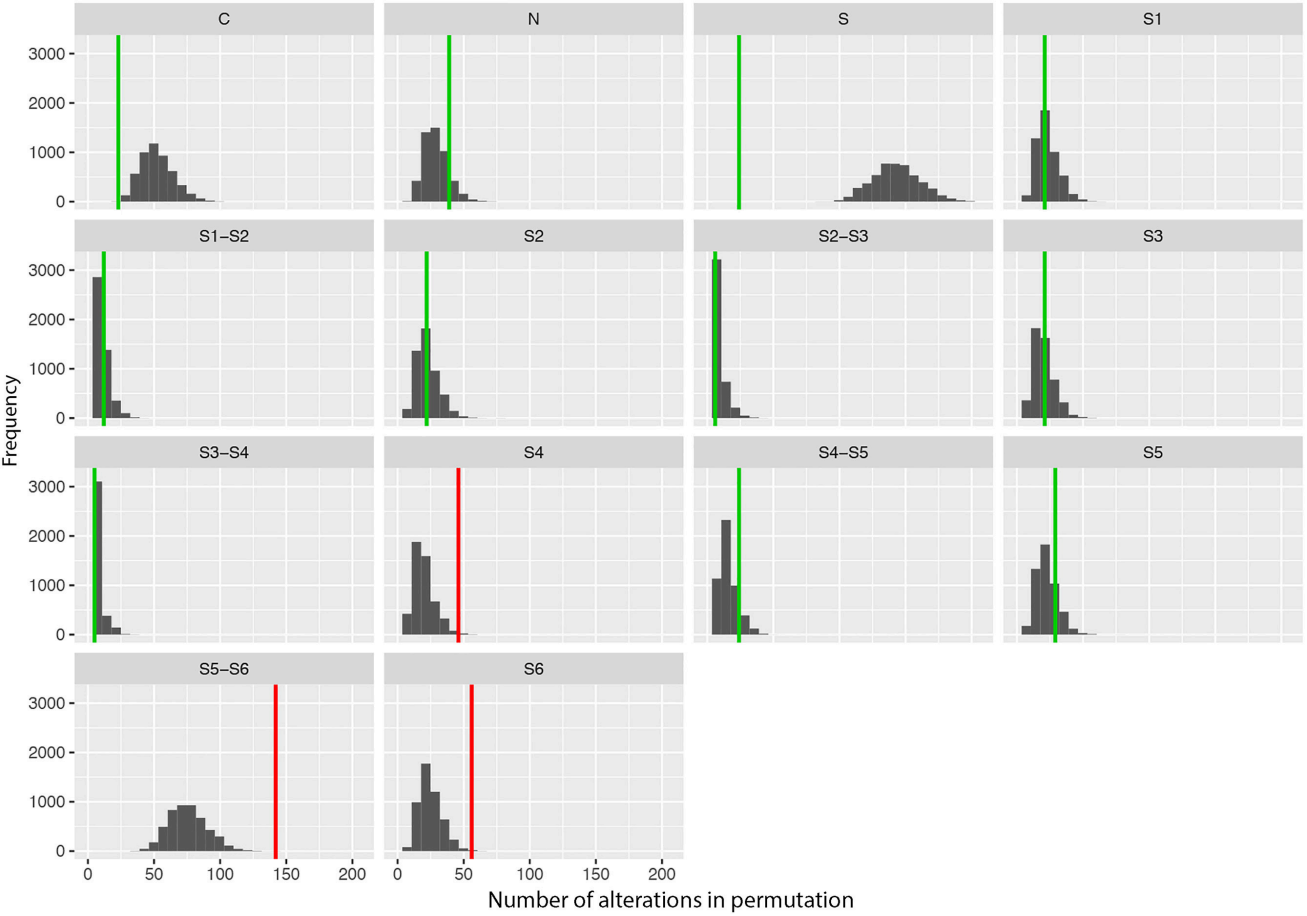
Results of the permutation test for the number of changes per segment showing the frequency of missense variants found in patients with Dravet syndrome per segment in 5,000 permutations of data. Vertical lines indicate the observed values (green: adjusted *p*-value α > 0.10; red: adjusted *p*-value α > 0.05).

We also performed a permutation analysis to test whether amino acid changes associated with DS were predominant in specific segments of the Nav1.1 protein. We found a clear predominance of amino acid changes in the voltage sensor segment (S4) and in the pore-forming region (S5–S6) and adjacent subunit S6. Permutation analysis showed that segments S4, S5–S6, and S6 presented more alterations than expected to occur by chance, with significant adjusted *P*-values (α < 0.05, Figure 2).

#### *In silico* Prediction Analysis

**Figure 3:**
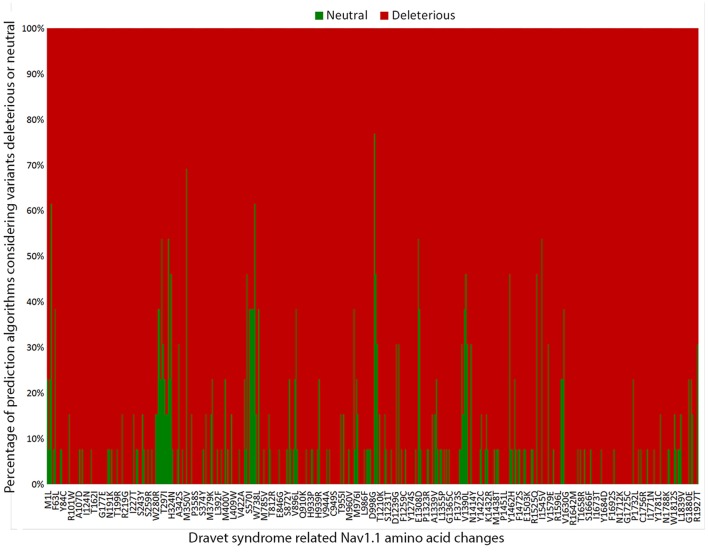
Graphic representation of the overall results of the 13 *in silico* prediction algorithms used to analyze 506 *SCN1A* missense mutations reported in patients with Dravet syndrome include in our metanalysis.

To estimate the deleterious effects of the 506 missense mutations reported in patients with DS, we used prediction algorithms separately for each variant. Our results showed that 98.4% (498/506) of amino acid changes are considered deleterious by more than half of the algorithms tested, and 65.2% (330/506) are predicted as deleterious by all 13 algorithms (Figure 3).

We applied the score classification workflow to all DS-related missense *SCN1A* variants (Table S3) and 56.5% (286/506) of the variants had their classification changed from VUS: 17.8% (90/506) into “pathogenic” and 38.7% (196/506) as “likely pathogenic.”

## Discussion

Even though there is a clear contribution of genetic testing to the establishment of more accurate diagnoses and a better understanding of the etiology of epilepsies, controversies regarding the clinical utility of genetic testing for different epilepsy syndromes have been frequently raised (1, 4, 30–32). Currently, one major limitation of the use of molecular testing in clinical practice is interpreting the biological relevance of variants found, as the causal relationship with the disease is not always evident, resulting in a dubious, erroneous or incomplete interpretation of the molecular results. Moreover, current guidelines state that VUS should not be used in clinical decision-making (3). Although the ACMG/AMP rules have improved clinical understanding of genetic data, there is still the need for specific guidelines in certain applications, as the number of VUS still restricts the proportions of conclusive diagnostic tests (3). In the present study, we advanced the classification of missense mutations found in one of the most relevant genes in the context of clinical testing in the epilepsies, *SCN1A*, by modifying the ACMG/AMP criteria and combining them into a classification workflow specific for DS.

From all the data gathered of *SCN1A* variants previously reported in the literature and our cohort, we observed that the most frequent type of mutations in patients with DS is the missense type (46%). Missense variants usually pose additional challenges regarding interpretation, as opposed to frameshift and nonsense mutations, which are often straightforwardly considered deleterious (3). This finding not only confirms that missense variants are frequent in patients with severe epilepsy phenotypes but also highlights the importance of advancing the knowledge available to perform the best possible clinical interpretation of this type of variant in the framework of genetic testing for epilepsy.

One strong evidence of pathogenicity is when an amino acid change has been already described as pathogenic (PS1). We found that only around 20% of the missense variants in *SCN1A* had been reported previously in more than one patient with DS in independent studies. Another strong evidence of pathogenicity is the presence of well-established functional studies showing a deleterious effect (PS3). However, we found few functional studies assessing the effects of DS-related amino acid changes in the Nav1.1 protein. Indeed, functional data are available for only 4.8% (24/502) of the amino acid changes reported. Therefore, by using these two current established strong criteria for pathogenicity < 25% of missense mutations would be classified as “pathogenic.”

We observed that the majority of *SCN1A* missense variants in DS are reported as *de novo*, 65.0% (329/506). However, if we consider only the variants with information available on the mode of inheritance, 86.6% (329/380) is *de novo* in at least one individual reported. We found that many studies do not disclose if the paternity was confirmed. Therefore, most variants would fulfill only the moderate evidence PM6 criteria, but not the strong evidence criteria PS2. By confirming the paternity in cases of *de novo* variant, the score of a variant could increase considerably. Unfortunately, there is still a lack of information on the mode of inheritance for 1/4 of the variants described. As it is an important criterion in the classification workflow, we strongly advocate the trio approach when performing genetic testing in patients with DS.

Furthermore, few studies show co-segregation of the disease associated *SCN1A* variant in multiple affected family members (PP1 criteria), and in most of these cases, the phenotype is not consistent with DS. Indeed, families segregating *SCN1A* mutations as an autosomal dominant trait have been reported (33, 34). In these families the clinical presentation is usually milder, which is more consistent with GEFS+ phenotype; however, some severe cases are also reported, underscoring the complex relationship between DS and GEFS+ (35–37).

We assessed the allele frequencies of the different variants found in patients with DS in genomic databases from different populations. The ACMG/AMP considers absence in population databases as moderate evidence of pathogenicity (PM2 criteria) (3). We observed that few variants (29/508 = 5.7%) were present in databases of control or reference individuals, thus indicating that the variants in *SCN1A* associated with DS are indeed rare but not absent in the general population. Only two variants found in patients (c.1811G>A and c.5782C>G) are present in all populations investigated and have allele frequencies higher than 1% in at least one of the population databases. Interestingly, a recent study critically re-evaluated these variants regarding their pathogenicity and considered them as benign (38). In addition, eight variants were identified in the heterozygous form in a single individual in a single population. This low frequency raises the possibility that an affected individual, especially one with a milder phenotype, could have been included in the population database inadvertently. Indeed, the curators of the gnomAD database (http://gnomad.broadinstitute.org/about) claim to have excluded individuals with severe pediatric disease and their relatives but also warn that some individuals with severe disease may still be included in the data set.

Another limitation of this type of analysis is that for some populations the databases are small (fewer than 100 individuals) and might not represent true population frequencies. In addition, populations of non-European ancestry are consistently underrepresented in population databases, which may significantly affect the interpretation of population frequencies of variants present in these individuals (39). Bias in the interpretation of variants in patients from non-European backgrounds may arise not only from an inaccurate estimation of allele frequencies of variants found in these patients but also from the possibility that variants present in low frequencies in these populations do not appear at all in the available databases, leading to a misdiagnosis of pathogenic variants. Given that *SCN1A* is a gene intolerant to variation, with a Z score for missense variants of 5.52 (deviation of observed counts from the expected number) published at the gnomAD database, the threshold for the maximum credible population allele frequency is expected to be low; thus, large data sets are required to perform an accurate estimation.

Furthermore, we observed a tendency toward clustering of putative pathogenic variants in specific regions/domains of the Nav1.1 protein. The segments S4, S5–S6, and S6, which comprise the voltage sensor and the pore-forming region of the protein, are more frequently affected by mutations. This supports the hypothesis that the location of missense mutations may contribute to the severity of the clinical phenotype. Since the ACMG guidelines indicate that location in a mutational hot spot and/or critical and well-established functional domain is considered moderate evidence of pathogenicity (PM1 criteria), we consider that *SCN1A* missense variants located in the segments S4, S5–S6, and S6 can be included in this category. However, despite this tendency for variants to concentrate in certain regions, they are still found throughout the entire protein.

Finally, we investigated the deleterious effects of the *SCN1A* missense mutations reported in patients with DS by using 13 prediction algorithms recommended by the ACMG/AMP guidelines. We found that over 98% (498/506) of *SCN1A* missense mutations in patients with DS were considered deleterious by more than half of the algorithms used, indicating that the mechanism most likely involved in the pathogenesis of DS by missense variants is indeed loss of protein function (38). All *in silico* programs agreed on the “pathogenic” prediction for 65.2% of the variants (330/506); thus, they fulfilled the PP3 criteria. Nevertheless, a few variants still showed conflicting prediction results in the *in silico* analysis. Therefore, one cannot completely rule out the possibility that variants predicted as benign represent non-deleterious *SCN1A* variants that were mistakenly implicated in the etiology of DS.

In conclusion, our results indicate that to better interpret the pathogenic impact of missense changes in *SCN1A* in the context of the molecular diagnosis of patients with DS, the use of multimodal analysis seems to be the best approach, since no single method can unequivocally classify all variants found. This includes assessment of information regarding previously established pathogenic amino acid changes and mode of inheritance, the determination of population-specific allele frequencies, localization within a specified protein domain and the use of different *in silico* prediction algorithms. The combination of these strategies seems to improve the accuracy of prediction and the likelihood of offering a clinically relevant report to clinicians as indicated by our results showing that by applying the proposed modified workflow, most DS related *SCN1A* variants had their classification changed from VUS to “pathogenic” or “likely pathogenic.”

## Data Availability

The datasets generated for this study can be found in The Brazilian Initiative on Precision Medicine (BIPMed), http://bipmed.iqm.unicamp.br/epileptic-encephalopathy.

## Author Contributions

MCG study concept and design, acquisition of data, analysis and interpretation of data, manuscript draft. MM, PP, MMG, and AC acquisition of data, critical revision of manuscript for intellectual content. MQ analysis and interpretation of data. BC analysis and interpretation of data, critical revision of manuscript for intellectual content. IL-C study concept and design, study supervision, critical revision of manuscript for intellectual content.

### Conflict of Interest Statement

IL-C received honorarium for participation as scientific advisor for BioMarin® in one occasion in December 2017. The remaining authors declare that the research was conducted in the absence of any commercial or financial relationships that could be construed as a potential conflict of interest

## Footnotes

^1^http://bipmed.iqm.unicamp.br/epileptic-encephalopathy

^2^https://www.ncbi.nlm.nih.gov/pubmed/

^3^cardiodb.org/allelefrequencyapp/

^4^http://bipmed.org/

^5^http://evs.gs.washington.edu/EVS/

^6^http://gnomad.broadinstitute.org/

^7^http://www.internationalgenome.org/

^8^http://fathmm.biocompute.org.uk

^9^http://bg.upf.edu/condel/home

^10^http://www.mutationtaster.org

^11^http://www.pantherdb.org/tools/csnpScoreForm.jsp

^12^http://snps.biofold.org/snps-and-go/snps-and-go.html

^13^http://mutpred.mutdb.org

^14^http://provean.jcvi.org/index.php

^15^http://cadd.gs.washington.edu

^16^http://genetics.bwh.harvard.edu/pph2/bgi.shtml

^17^http://mutationassessor.org/r3

^18^http://siftdna.org/www/SIFT_pid_subst_all_submit.html

^19^http://agvgd.hci.utah.edu/agvgd_input.php

^20^http://snps.biofold.org/phd-snp/phd-snp.html
